# Ulcère de jambe révélant la leishmaniose cutanée

**DOI:** 10.11604/pamj.2017.27.287.13090

**Published:** 2017-08-23

**Authors:** Elalouani Elmehdi, Bachar Zerkly

**Affiliations:** 1 Service de Traumatologie Orthopedie du Groupe Hospitalier Publique du Sud de l’Oise(GHPSO), bp60100 Creil, France

**Keywords:** Ulcère de jambe, leishmaniose cutanée, L. major, leg ulcer, cutaneous leishmaniasis, L major

## Abstract

Leishmaniases are parasitic diseases occurring in endemic tropical and subtropical areas and caused by protozoa of the genus leishmania, transmitted by a diptera (sand fly). We here report a case of topical cutaneous leishmaniasis discovered in a 15-year old boy with painless ulcer on his left leg, who had been staying in South Africa. Clinical examination showed painless non-itchy ulcer, occurred 1 month before, on the antero-internal part of his left leg with crusts and scars caused by insect bites, all evolving in a context of patient’s general health status, without mucosal or visceral lesions. Skin biopsy allowed specific parasitologic diagnosis revealing topical zoonotic cutaneous leishmaniasis caused by L. major. The patient underwent topical treatment based on paramomycin and oral fluconazole resulting in ulcer healing at the end of 2 months.

## Image en médecine

Les leishmanioses sont des parasitoses présentes dans les zones d'endémie tropicales et subtropicales dues à des protozoaires du genre leishmania, transmises par un diptère (phlébotome). Nous présentons le cas d'une leishmaniose cutanée localisée découverte chez un enfant de 15 ans ayant séjourner en Afrique du sud, présentant un ulcère indolore de la jambe gauche. Cliniquement: l'ulcère est indolore remonte à un mois, non prurigineux siégeant sur la face antéro interne de la jambe gauche avec présence de croutes et des cicatrise de piqure des insectes le tout évoluant dans un contexte de conservation de l'état général, sans lésion muqueuse ni viscérale. Diagnostic spécifique parasitologique est mis évidence par la biopsie cutanée, révélant une leishmaniose cutanée localisée zoonotique à L. major. L'enfant a été traité par traitement local par paramomycine et fluconazole oral avec cicatrisation de l'ulcère au bout de 2 mois.

**Figure 1:**
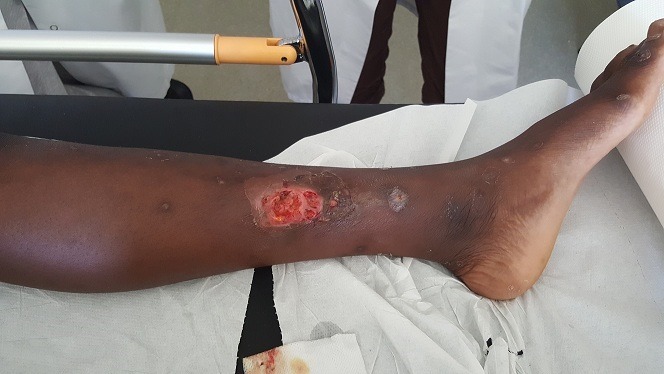
Image clinique d’un un ulcère indolore de la jambe gauche chez un jeune patient de 15 ayant séjourner en Afrique du sud, révélant une leishmaniose cutanée localisé

